# Supplementary material for: Mlp1 and Mlp2 cooperate to build a stoichiometric nuclear pore basket in budding yeast
Source: J Cell Sci. 2026 Jul 27;139(14):jcs264865. doi: 10.1242/jcs.264865 (PMC13490735; doi:10.1242/jcs.264865)
Supplement: Supplementary file 1 [file joces-139-264865-s1.pdf]

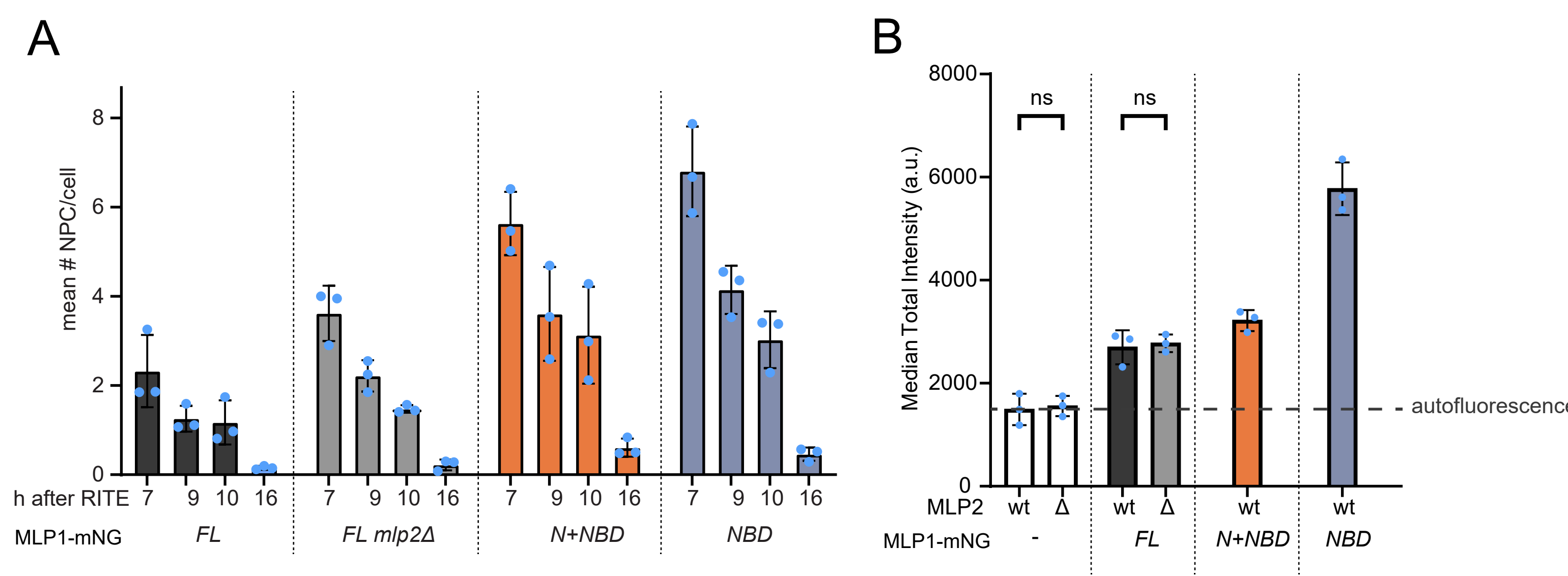

Fig. S1. Mlp1 truncations bind the NPC stably.

(A) Quantification of foci (single NPCs) in doRITE assay with Mlp1 truncation constructs (compare Figure 1D). Dots were manually counted in z stacks of 100 randomly chosen cells per time point and replicate. Blue dots represent biological replicates. Error bars represent the standard deviation.

(B) Total fluorescence intensity of cells shown in Figure 1D at the 0 h timepoint. Blue dots represent biological replicates. Error bars represent the standard deviation. wt and *mlp2Δ* were not significantly different by paired two-tailed t-test (ns).

Journal of Cell Science • Supplementary information

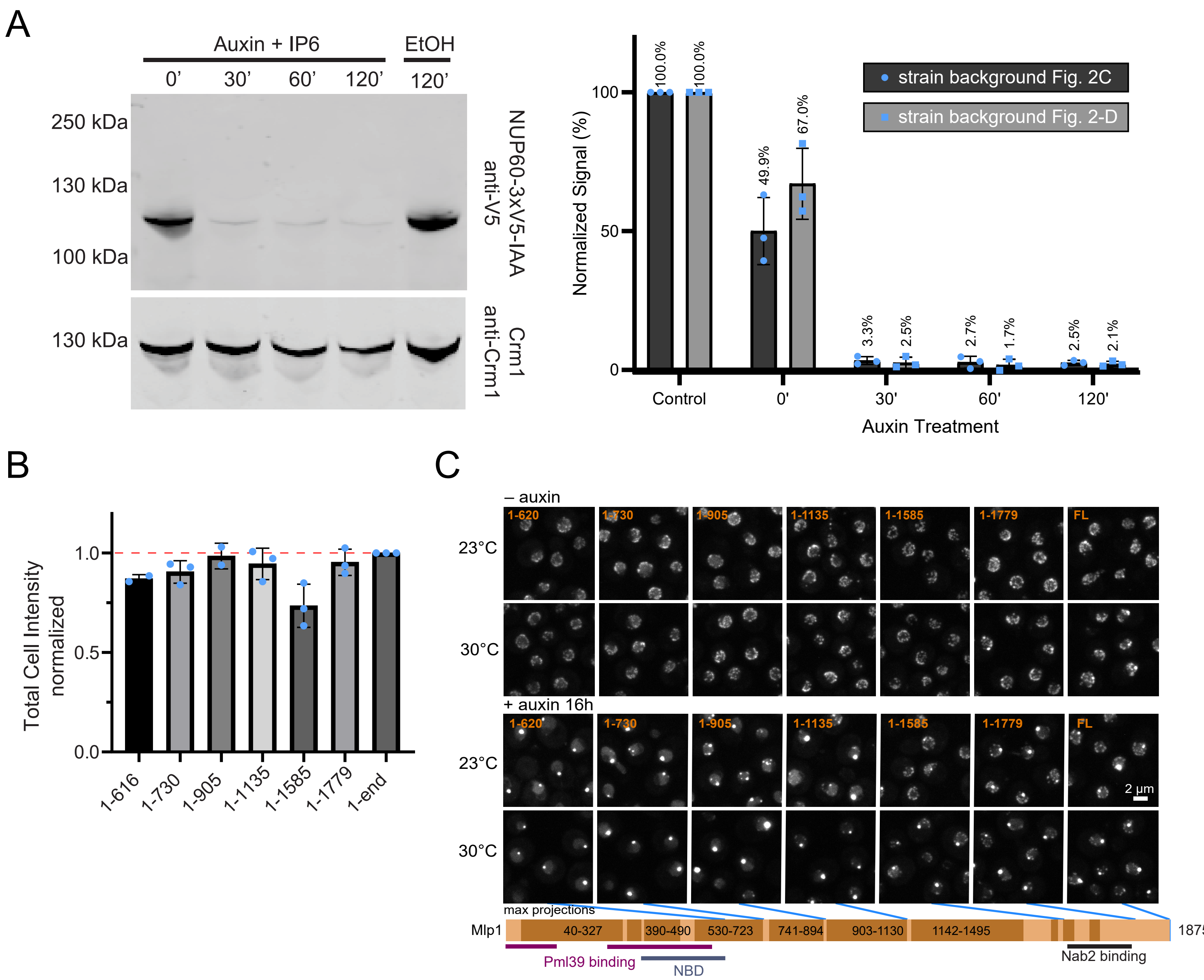

Fig. S2. The Mlp1 C-terminus confers Nup60-independent binding at the NPC.

(A) Western blot showing depletion of Nup60 upon addition of auxin. Example blot of the strain background for the strains in Figure 2C is shown. Quantification on the right shows biological triplicates for the two strain backgrounds used in Figure 2C and 2D&S2B.

(B) Total fluorescence intensity of cells expressing different C-terminal truncations of Mlp1 in the absence of auxin to compare expression levels. Total fluorescence intensity was normalized to the full-length construct. Blue dots represent biological replicates. Bars show the mean, error bars represent the standard deviation.

(C) Localization of NLS-EGFP tagged, C-terminally truncated versions of Mlp1 after overnight growth in the absence of Nup60 by auxin-induced depletion at either 23 °C or 30 °C.

Image panels shown are maximum intensity projections.

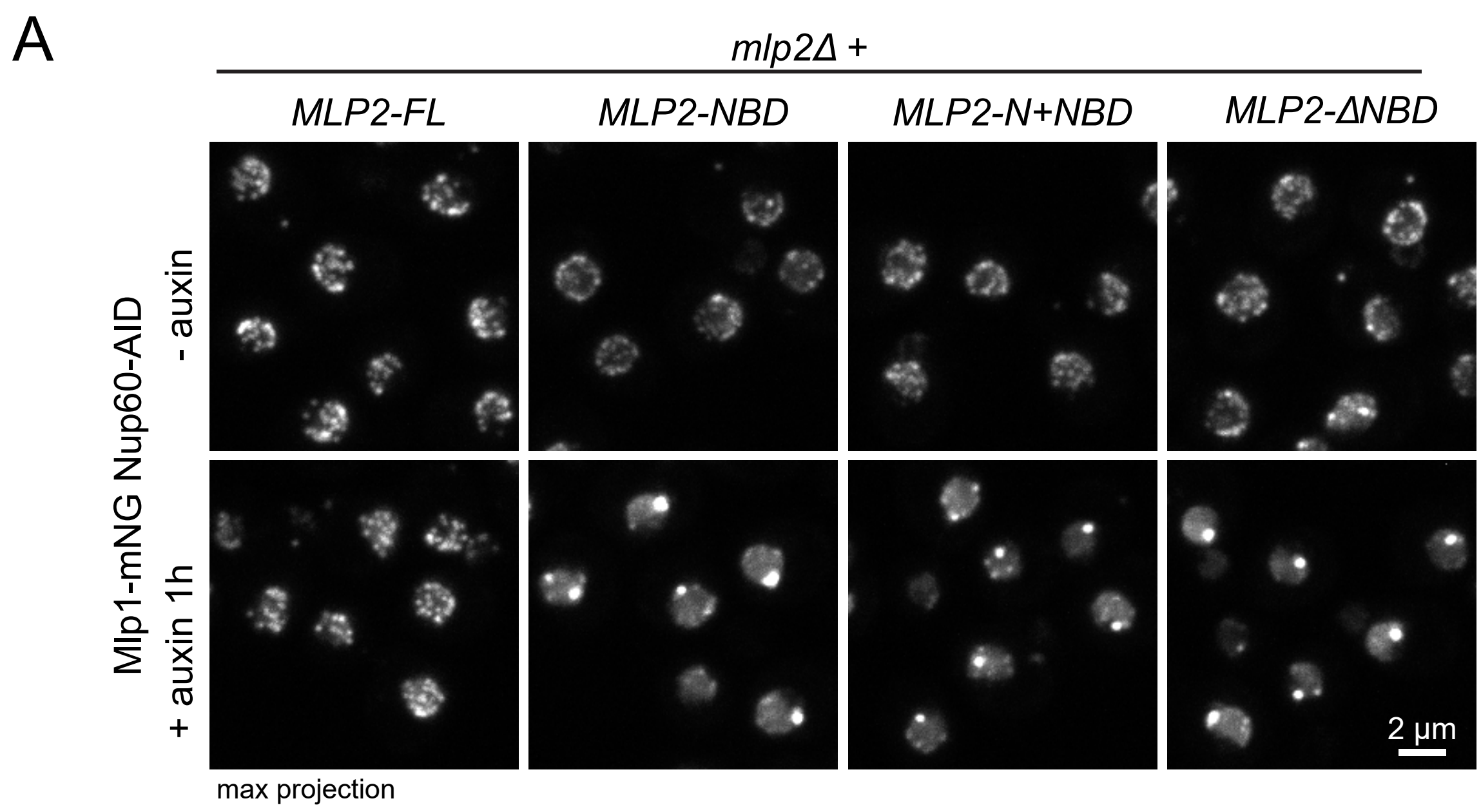

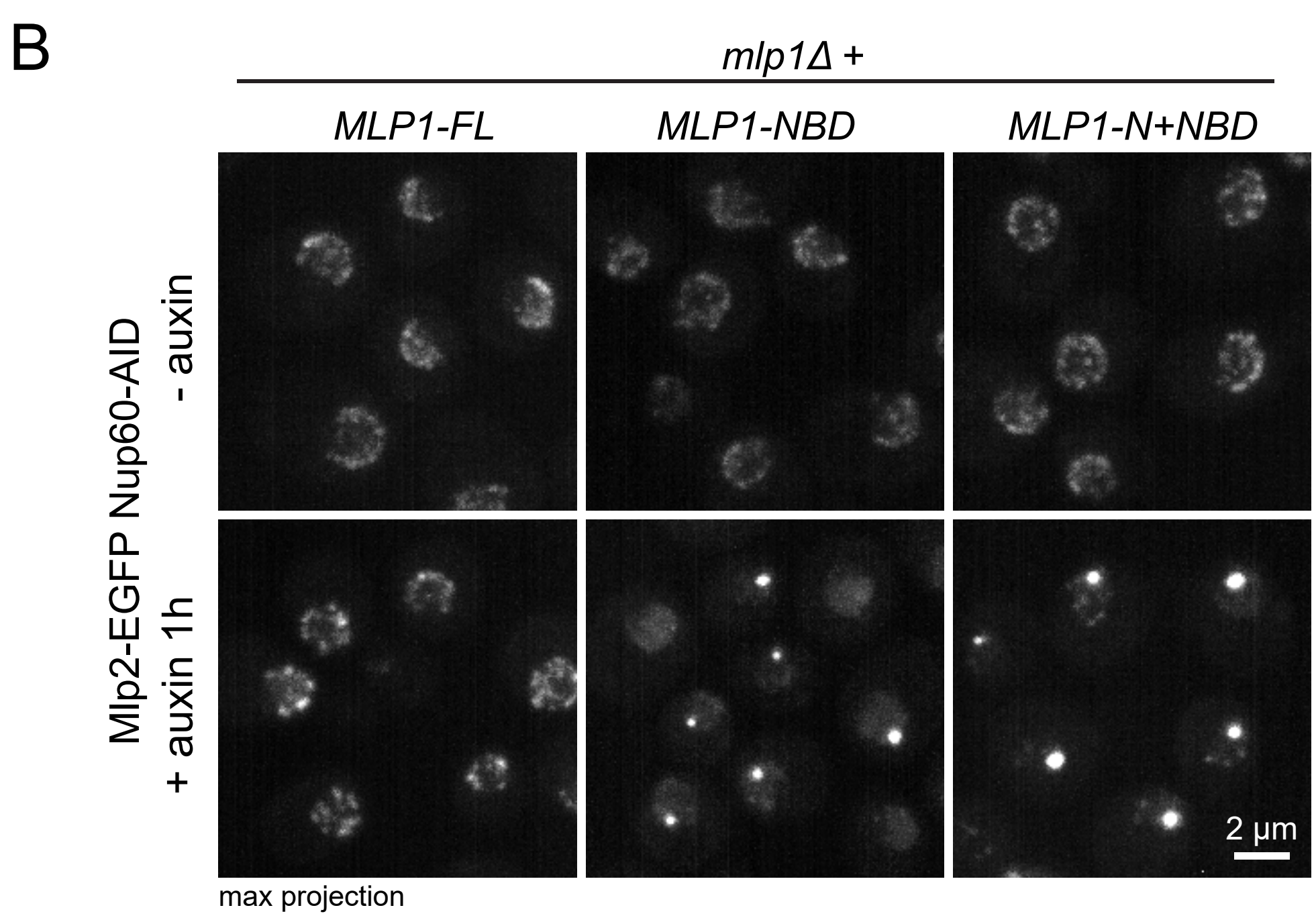

Fig. S3. Mlp1 and Mlp2 stabilize each other at the NPC in the absence of Nup60.

(A) Localization of Mlp1-mNG upon rescue of *mlp2Δ* with different Mlp2 truncation constructs. Cells were treated with auxin for 1h to deplete Nup60.

(B) Localization of Mlp2-mNG upon rescue of with different Mlp1 truncation constructs. Cells were treated with auxin for 1h to deplete Nup60.

Journal of Cell Science • Supplementary information

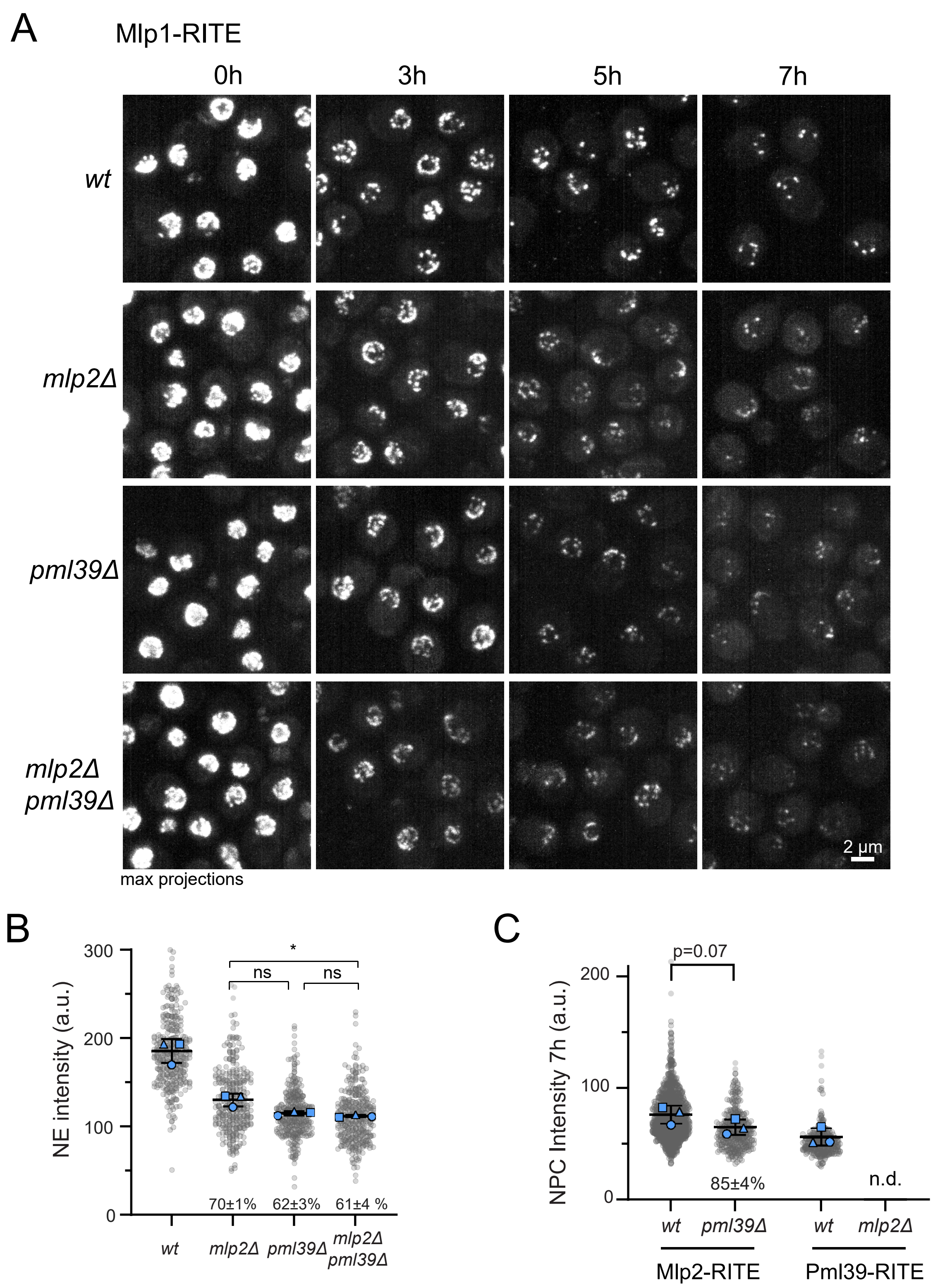

Fig. S4. Mlp2 contributes to anchoring of Mlp1 to the NPC.

(A) Additional timepoints for the RITE time course shown in Figure 4A. Maximum intensity projections are shown.

(B) Quantification of NE intensity at 0h timepoint for data shown in Figure 4A. Grey circles represent background-subtracted measurements from individual cells. Blue symbols represent means of individual biological replicates. Error bars represent the standard deviation. Statistical analysis was carried out a repeated measures one-way ANOVA with Tukey's multiple comparison test. ns: not significant, * $p < 0.05$. All other comparison (wt with each mutant) are significant with $p \leq 0.0001$. Percentage values indicate the relative mean intensity compared to wt ± standard deviation.

(C) Quantification of mean NPC intensity at the 7h timepoint for data shown in Figure 4C and 4D. Grey circles represent background-subtracted measurements from >75 individual NPCs per biological replicate. Blue symbols represent means of individual biological replicates. Error bars represent the standard deviation. p value is for a two-tailed paired t-test. Percentage value indicates the relative mean intensity compared to wt ± standard deviation.

Journal of Cell Science • Supplementary information

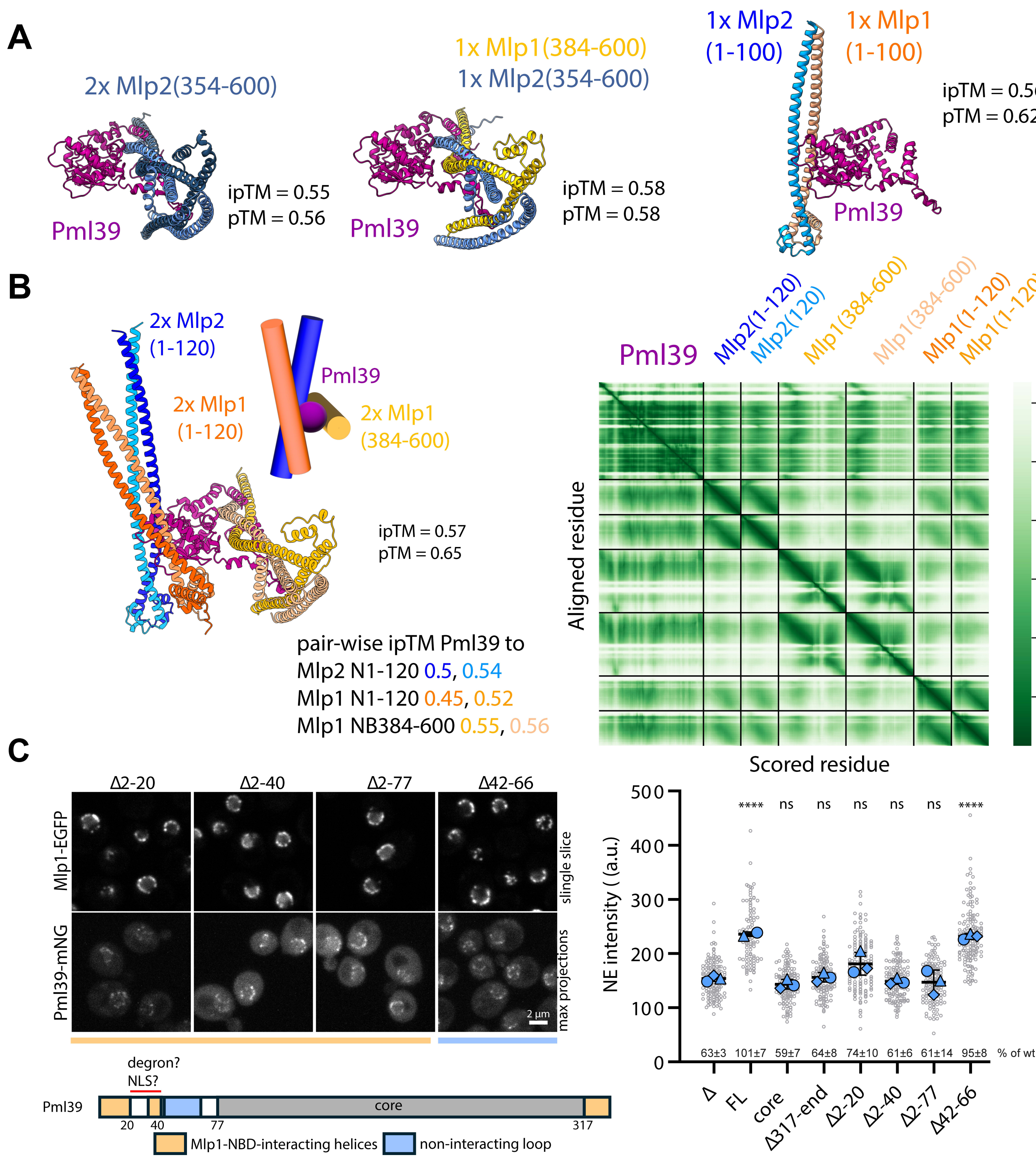

Fig. S5. Investigating the interaction network of Pml39 at the NPC.

(A) AlphaFold3 model of the interaction of Pml39 with the NPC binding domain of Mlp1 and the N-termini of Mlp1 and Mlp2. Overall AlphaFold scores are given.

(B) AlphaFold3 model of the interaction of Pml39 with the NPC binding domain of Mlp1 and the N-termini of Mlp1 and Mlp2. Schematic on top right illustrates orientation of proteins toward each other. The matrices of the PAE scores is shown on the right. The overall ipTM and pTM scores as well as the pairwise ipTM scores are shown below.

(C) Localization of Mlp1-EGFP in strains expressing additional Pml39 truncation mutants. Plot shows quantification of Mlp1-EGFP intensity at the nuclear envelope. Grey circles represent background-subtracted measurements from individual cells (~50 cells per condition and biological replicate). Blue symbols represent means of individual biological replicates. Error bars represent the standard deviation. Statistical testing was performed by fitting a mixed effects model with Sidak's multiple comparison test comparing each strain to Δ. **** < 0.0001 , ns > 0.05. Percentage values indicate the relative mean intensity compared to FL ± standard deviation. Note that the quantification data for the strains shown in Fig. 5E is shown again for comparison. Schematic indicates the different deleted regions on the Pml39 architecture. A region that appears to affect the expression level of the truncation mutants is indicated in red.

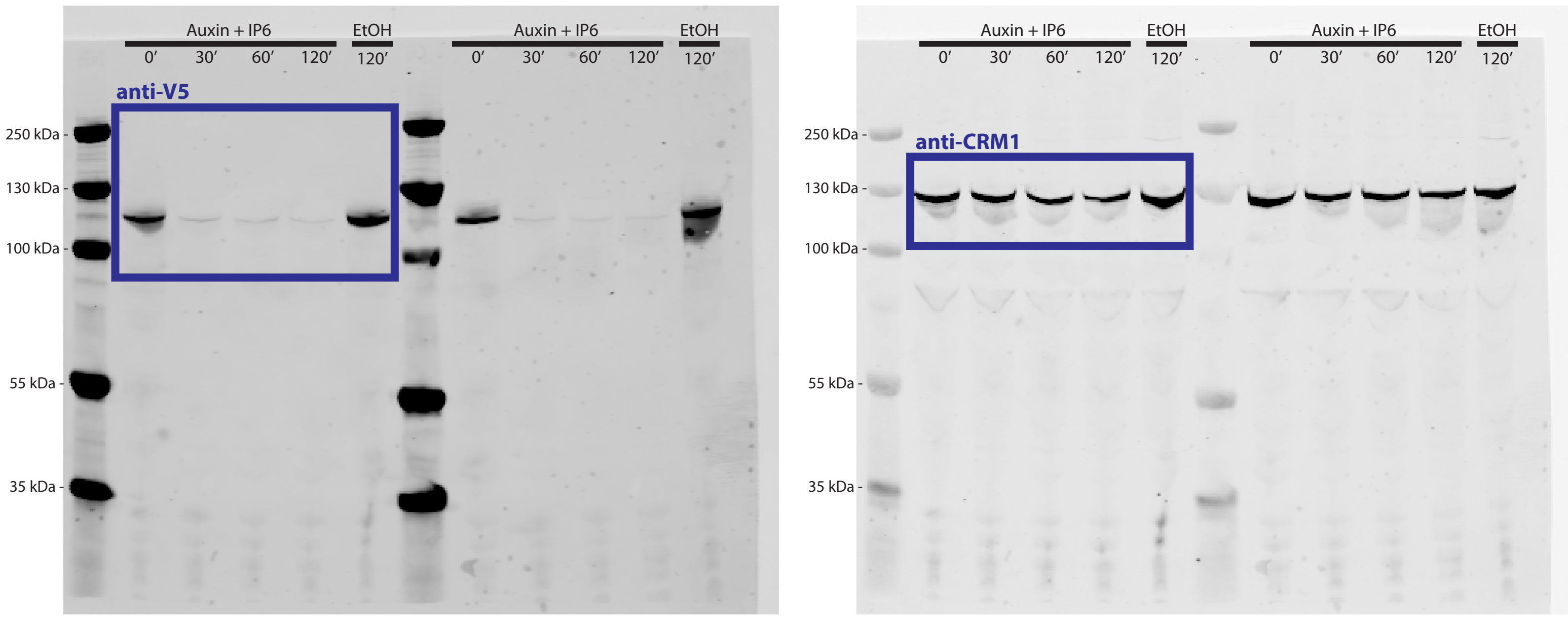

Fig. S6. Blot Transparency.
Full Western blots of blots used in Fig.S2A are shown.

Journal of Cell Science • Supplementary information

Table S1. Yeast strains

Strains	Genotype	used in Fig.	Reference
KWY1601	BY4741: MAT A his3Δ1 leu2Δ0 ura3Δ0 met15Δ0	parental strain	Brachmann et al. 1998
KWY1602	BY4742: MAT α his3Δ1 leu2Δ0 ura3Δ0 lys2Δ0	parental strain	Brachmann et al. 1998
KWY12605	MAT A mlp1Δ::KanMx HIS3::CRE-EBD TRP1::pMlp1-Mlp1(384-616)-loxP- mNG_URA3-loxP_nothing	1D, S1A, S1B	this work
KWY12606	MAT A mlp1Δ::KanMx HIS3::CRE-EBD TRP1::pMlp1-Mlp1(1-620)-loxP- mNG_URA3-loxP_nothing	1D, S1A, S1B	this work
KWY12842	MAT A mlp1Δ::KanMx HIS3::CRE-EBD TRP1::pMlp1-Mlp1-loxP- mNG_URA3-loxP_nothing	1D, S1A, S1B	this work
KWY12843	MAT A mlp1Δ::KanMx HIS3::CRE-EBD TRP1::pMlp1-Mlp1-loxP- mNG_URA3-loxP_nothing mlp2Δ::NatMx	1D, S1A, S1B	this work
KWY12371	MAT A mlp1Δ::KanMx HIS3::CRE-EBD	S1B	this work
KWY12372	MAT A mlp1Δ::KanMx HIS3::CRE-EBD mlp2Δ::NatMx	S1B	this work
KWY10385	MAT A Mlp1(1-616)-SV40NLS-EGFP::URA3	2B	this work
KWY10438	MAT A Mlp1(1-616)-SV40NLS-EGFP::URA3 nup60Δ::KanMx	2B	this work
KWY12166	MAT α MLP1::MLP1-loxP-GFP_URA3-loxP_nothing HIS3::CRE-EBD nup60Δ::KanMx	2B	this work
KWY10309	MAT α MLP1::MLP1-loxP-GFP_URA3-loxP_nothing HIS3::CRE-EBD	2B, 4A, S4A, S4B	Zsok et al., 2024
KWY12584	MAT α NUP60::NUP60-3xV5-IAA-NatMX mlp1Δ::KanMx TRP1::osTIR-Tadh1-HPHMX LEU2::pMLP1-SV40NLS-MLP1(aa1-620)-mNeonGreen	2C	this work
KWY12611	MAT α NUP60::NUP60-3xV5-IAA-NatMX mlp1Δ::KanMx TRP1::osTIR-Tadh1-HPHMX LEU2::pMLP1-SV40NLS-MLP1(aa385-616)-mNeonGreen	2C	this work
KWY12844	MAT α NUP60::NUP60-3xV5-IAA-NATMX mlp1Δ::KanMx TRP1::osTIR-Tadh1-HPHMX LEU2::pMLP1-SV40NLS-MLP1-mNeonGreen	2C, 3C	this work
KWY4374	NUP60::NUP60-3xV5-IAA-NATMX TRP1::osTIR-Tadh1-HPHMX MLP1(1-616)::-SV40NLS-EGFP::CaURA3	2D, S2B, S2C	this work
KWY4380	NUP60::NUP60-3xV5-IAA-NATMX TRP1::osTIR-Tadh1-HPHMX MLP1(1-905)::-SV40NLS-EGFP::CaURA3	2D, S2B, S2C	this work
KWY4391	NUP60::NUP60-3xV5-IAA-NATMX TRP1::osTIR-Tadh1-HPHMX MLP1(1-730)::-SV40NLS-EGFP::CaURA3	2D, S2B, S2C	this work
KWY4393	NUP60::NUP60-3xV5-IAA-NATMX TRP1::osTIR-Tadh1-HPHMX MLP1(1-1135)::-SV40NLS-EGFP::CaURA3	2D, S2B, S2C	this work
KWY4417	NUP60::NUP60-3xV5-IAA-NATMX TRP1::osTIR-Tadh1-HPHMX MLP1::-SV40NLS-EGFP::CaURA3	2D, S2B, S2C	this work
KWY4418	NUP60::NUP60-3xV5-IAA-NATMX TRP1::osTIR-Tadh1-HPHMX MLP1(1-1489)::-SV40NLS-EGFP::CaURA3	2D, S2B, S2C	this work
KWY4419	NUP60::NUP60-3xV5-IAA-NATMX TRP1::osTIR-Tadh1-HPHMX MLP1(1-1585)::-SV40NLS-EGFP::CaURA3	2D, S2B, S2C	this work
KWY4420	NUP60::NUP60-3xV5-IAA-NATMX TRP1::osTIR-Tadh1-HPHMX MLP1(1-1779)::-SV40NLS-EGFP::CaURA3	2D, S2B, S2C	this work
KWY12373	MAT α NUP60::NUP60-3xV5-IAA-NatMX mlp1Δ::KanMx TRP1::osTIR-Tadh1-HPHMX	S2A	this work
KWY12580	NUP60::NUP60-3xV5-IAA-NatMX TRP1::osTIR-Tadh1-HPHMX	S2A	this work
KWY12935	MAT A his3Δ1 leu2Δ0 lys2Δ0 ura3Δ0 mlp1Δ::KanMx mlp2Δ::NatMx URA3::pMLP1-SV40NLS-MLP2-mNG	3B	this work
KWY12936	MAT A his3Δ1 leu2Δ0 lys2Δ0 ura3Δ0 mlp1Δ::KanMx mlp2Δ::NatMx URA3::pMLP1-SV40NLS-MLP2(aa354-600)-mNG	3B	this work
KWY13029	mlp2Δ::NatMx URA3::SV40NLS-Mlp2FL-mNG::URA3	3B	this work
KWY13030	mlp2Δ::NatMx URA3::SV40NLS-Mlp2NBD-mNG::URA3	3B	this work
KWY13032	mlp2Δ::NatMx URA3::SV40NLS-Mlp2ΔNBD-mNG::URA3	3B	this work
KWY13033	MAT A his3Δ1 leu2Δ0 lys2Δ0 ura3Δ0 mlp1Δ::KanMx mlp2Δ::NatMx URA3::SV40NLS-MLP2ΔNBD-mNG	3B	this work
KWY13005	MAT α NUP60::NUP60-3xV5-IAA-NATMX mlp1Δ::KanMx TRP1::osTIR-Tadh1-HPHMX LEU2::pMLP1-SV40NLS-MLP1-mNeonGreen mlp2Δ::His3	3C	this work
KWY12970	NUP60::NUP60-3xV5-IAA-NATMX TRP1::osTIR-Tadh1-HPHMX MLP2::-EGFP;URA3	3D	this work
KWY13004	NUP60::NUP60-3xV5-IAA-NATMX TRP1::osTIR-Tadh1-HPHMX MLP2::-EGFP;URA3 mlp1Δ::KanMx	3D	this work
KWY12969	MAT α MLP2::MLP2-loxP-GFP_URA3-loxP_nothing HIS3::CRE-EBD mlp1Δ::NatMX	3E	this work
KWY10750	MAT α MLP2::MLP2-loxP-GFP_URA3-loxP_nothing HIS3::CRE-EBD	3E, 4C, S4C	Zsok et al., 2024
KWY13008	MAT α NUP60::NUP60-3xV5-IAA-NATMX mlp1Δ::KanMx TRP1::osTIR-Tadh1-HPHMX LEU2::pMLP1-SV40NLS-MLP1-mNeonGreen mlp2Δ::His3 URA3::SV40NLS-Mlp2FL-3xHA	S3A	this work
KWY13009	MAT α NUP60::NUP60-3xV5-IAA-NATMX mlp1Δ::KanMx TRP1::osTIR-Tadh1-HPHMX LEU2::pMLP1-SV40NLS-MLP1-mNeonGreen mlp2Δ::His3 URA3::SV40NLS-Mlp2NBD-3xHA	S3A	this work
KWY13010	MAT α NUP60::NUP60-3xV5-IAA-NATMX mlp1Δ::KanMx TRP1::osTIR-Tadh1-HPHMX LEU2::pMLP1-SV40NLS-MLP1-mNeonGreen mlp2Δ::His3 URA3::SV40NLS-Mlp2N+NBD-3xHA	S3A	this work
KWY13034	MAT α NUP60::NUP60-3xV5-IAA-NATMX mlp1Δ::KanMx TRP1::osTIR-Tadh1-HPHMX LEU2::pMLP1-SV40NLS-MLP1-mNeonGreen mlp2Δ::His3 URA3::SV40NLS-Mlp2ΔNBD-3xHA	S3A	this work
KWY13025	NUP60::NUP60-3xV5-IAA-NATMX TRP1::osTIR-Tadh1-HPHMX MLP2::-EGFP;URA3 mlp1Δ::KanMx trp1Δ::SV40NLS-Mlp1NBD-3xHA;LEU2	S3B	this work
KWY13026	NUP60::NUP60-3xV5-IAA-NATMX TRP1::osTIR-Tadh1-HPHMX MLP2::-EGFP;URA3 mlp1Δ::KanMx trp1Δ::SV40NLS-Mlp1N+NBD-3xHA;LEU2	S3B	this work

KWY13027	NUP60::NUP60-3xV5-IAA-NATMX TRP1::osTIR-Tadh1-HPHMX MLP2::-EGFP;URA3 mlp1Δ::KanMx trp1Δ::SV40NLS-Mlp1FL-3xHA;LEU2	S3B	this work
KWY12230	MAT A MLP1::MLP1-loxP-GFP_URA3-loxP_nothing HIS3::CRE-EBD mlp2Δ::NatMX	4A, S4A, S4B	this work
KWY4402	MAT A MLP1::MLP1-loxP-GFP_URA3-loxP_nothing HIS3::CRE-EBD mlp2Δ::NatMX pml39Δ::KanMX	4A, S4A, S4B, 5D	this work
KWY4403	MAT α PML39::PML39-loxP-GFP_URA3-loxP_nothing HIS3::CRE-EBD mlp2Δ::KanMX	4A, S4A, S4B, 5D	this work
KWY10882	MAT α MLP1::MLP1-loxP-GFP_URA3-loxP_nothing HIS3::CRE-EBD pml39Δ::NatMX	4A, S4A, S4B, 5E, S5C	Zsok et al., 2024
KWY4404	MAT α MLP2::MLP2-loxP-GFP_URA3-loxP_nothing HIS3::CRE-EBD pml39Δ::KanMX	4C, S4C	this work
KWY10998	MAT α PML39::PML39-loxP-GFP_URA3-loxP_nothing HIS3::CRE-EBD	4D, S4C	Zsok et al., 2024
KWY12874	MAT α mlp1Δ::KanMx Pml39-yEGFP::URA3	5C	this work
KWY13040	MAT α mlp1Δ::KanMx Pml39-yEGFP::URA3 trp1Δ::SV40NLS-MLP1-3xHA;LEU2	5C	this work
KWY13041	MAT α mlp1Δ::KanMx Pml39-yEGFP::URA3 trp1Δ::SV40NLS-MLP1ΔN1-70-3xHA;LEU2	5C	this work
KWY13042	MAT α mlp1Δ::KanMx Pml39-yEGFP::URA3 LEU2::SV40NLS-MLP1N+NBD-3xHA	5C	this work
KWY13043	MAT α mlp1Δ::KanMx Pml39-yEGFP::URA3 LEU2::SV40NLS-MLP1NBD-3xHA	5C	this work
KWY13011	MAT α PML39::PML39-loxP-GFP_URA3-loxP_nothing HIS3::CRE-EBD mlp2Δ::KanMX trp1Δ::SV40NLS-Mlp2FL-3xHA;LEU2	5D	this work
KWY13012	MAT α PML39::PML39-loxP-GFP_URA3-loxP_nothing HIS3::CRE-EBD mlp2Δ::KanMX trp1Δ::SV40NLS-Mlp2NBD-3xHA;LEU2	5D	this work
KWY13013	MAT α PML39::PML39-loxP-GFP_URA3-loxP_nothing HIS3::CRE-EBD mlp2Δ::KanMX trp1Δ::SV40NLS-Mlp2N+NBD-3xHA;LEU2	5D	this work
KWY13014	MAT α PML39::PML39-loxP-GFP_URA3-loxP_nothing HIS3::CRE-EBD mlp2Δ::KanMX trp1Δ::SV40NLS-Mlp2ΔN1-70-3xHA;LEU2	5D	this work
KWY12891	MAT α HIS3::CRE-EBD MLP1::MLP1-3xV5 pml39Δ::NatMX	5E, S5C	this work
KWY12931	HIS3::CRE-EBD MLP1::MLP1-loxP-GFP_URA3-loxP_nothing pml39Δ::NatMX LEU2::pMLP1-PML39-3xHA	5E, S5C	this work
KWY12933	MAT α HIS3::CRE-EBD MLP1::MLP1-3xV5 pml39Δ::NatMX LEU2::pMLP1-Pml39-mNG	5E, S5C	this work
KWY13019	MAT α HIS3::CRE-EBD MLP1::MLP1-3xV5 pml39Δ::NatMX trp1Δ::pMLP1-Pml39core(aa78-316)-mNG;LEU2	5E, S5C	this work
KWY13024	HIS3::CRE-EBD MLP1::MLP1-loxP-GFP_URA3-loxP_nothing pml39Δ::NatMX trp1Δ::pMLP1-PML39core(aa78-316)-3xHA;LEU2	5E, S5C	this work
KWY13037	MAT α HIS3::CRE-EBD MLP1::MLP1-3xV5 pml39Δ::NatMX trp1Δ::pMLP1-Pml39Δ317-end-mNG;LEU2	5E, S5C	this work
KWY13015	MAT α HIS3::CRE-EBD MLP1::MLP1-3xV5 pml39Δ::NatMX trp1Δ::pMLP1-Pml39Δ3-20-mNG;LEU2	S5C	this work
KWY13016	MAT α HIS3::CRE-EBD MLP1::MLP1-3xV5 pml39Δ::NatMX trp1Δ::pMLP1-Pml39Δ3-40-mNG;LEU2	S5C	this work
KWY13017	MAT α HIS3::CRE-EBD MLP1::MLP1-3xV5 pml39Δ::NatMX trp1Δ::pMLP1-Pml39Δ3-77-mNG;LEU2	S5C	this work
KWY13020	HIS3::CRE-EBD MLP1::MLP1-loxP-GFP_URA3-loxP_nothing pml39Δ::NatMX trp1Δ::pMLP1-PML39Δ3-20-3xHA;LEU2	S5C	this work
KWY13021	HIS3::CRE-EBD MLP1::MLP1-loxP-GFP_URA3-loxP_nothing pml39Δ::NatMX trp1Δ::pMLP1-PML39Δ3-40-3xHA;LEU2	S5C	this work
KWY13022	HIS3::CRE-EBD MLP1::MLP1-loxP-GFP_URA3-loxP_nothing pml39Δ::NatMX trp1Δ::pMLP1-PML39Δ3-77-3xHA;LEU2	S5C	this work
KWY13036	MAT α HIS3::CRE-EBD MLP1::MLP1-3xV5 pml39Δ::NatMX trp1Δ::pMLP1-Pml39Δ42-66-mNG;LEU2	S5C	this work
KWY13038	HIS3::CRE-EBD MLP1::MLP1-loxP-GFP_URA3-loxP_nothing pml39Δ::NatMX trp1Δ::pMLP1-PML39Δ42-66-3xHA;LEU2	S5C	this work
KWY13039	HIS3::CRE-EBD MLP1::MLP1-loxP-GFP_URA3-loxP_nothing pml39Δ::NatMX trp1Δ::pMLP1-PML39Δ317-end-3xHA;LEU2	S5C	this work

Journal of Cell Science • Supplementary information

Table S2. Plasmids

**Yeast Integration Plamids**		**Reference**
pKW3061	pRS303-GPD-CRE-EDB78	Terweij et al., 2013
pKW4362	pGPD-osTIR-hphNT1-TRP1	Heinrich et el., 2024
pKW6287	pMLP1-SV40NLS-Mlp1(AA1-620)-mNeonGreen-tADH1-LEU2integration	this work
pKW6300	pMLP1-SV40NLS-Mlp1(AA1-620)-RITE(mNG-to-dark-URA3)-tADH1-TRP1integration	this work
pKW6301	pMLP1-SV40NLS-Mlp1(AA385-616)-RITE(mNG-to-dark-URA3)-tADH1-TRP1integratio	this work
pKW6304	pMLP1-SV40NLS-Mlp1(AA385-616)-mNeonGreen-tADH1-LEU2integration	this work
pKW6419	pMLP1-SV40NLS-Mlp1-mNG;LEU2_LEU2 integration	this work
pKW6421	pMLP1-SV40NLS-Mlp1-mCherry;LEU2_LEU2 integration	this work
pKW6426	pMLP1-SV40NLS-Mlp1-RITE(mNG-to-dark-URA3)-tADH1-TRP1integration	this work
pKW6427	pMLP1-SV40NLS-Mlp1(1-620)-mCherry;LEU2_LEU2 integration	this work
pKW6428	pMLP1-SV40NLS-Mlp1(385-616)-3xHA;LEU2_LEU2 integration	this work
pKW6484	pMLP1-PML39-3xHA_LEU2 integration	this work
pKW6485	pMLP1-PML39core(aa77-317)-3xHA_LEU2 integration	this work
pKW6491	pMLP1-PML39core(aa77-317)-mNeonGreen_LEU2 integration	this work
pKW6501	pMLP1-SV40NLS-MLP2-mNG_URA integration	this work
pKW6502	pMLP1-SV40NLS-MLP2(aa354-600)-mNG_URA integration	this work
pKW6590	pMLP1-PML39-mNeonGreen_LEU2 integration	this work
pKW6635	pMLP1-SV40NLS-Mlp1-FL-3xHA; LEU2; TRP1 integration	this work
pKW6636	pMLP1-SV40NLS-Mlp2-FL-3xHA; LEU2; TRP1 integration	this work
pKW6637	pMLP1-SV40NLS-Mlp1-ΔN1-70-3xHA; LEU2; TRP1 integration	this work
pKW6638	pMLP1-SV40NLS-Mlp2-ΔN1-70-3xHA; LEU2; TRP1 integration	this work
pKW6641	pMLP1-SV40NLS-Mlp2-N+NBD(aa1-600)-mNG; URA3 integration	this work
pKW6642	pMLP1-SV40NLS-Mlp2-FL-3xHA; URA3 integration	this work
pKW6643	pMLP1-SV40NLS-Mlp2-NBD(aa354-600)-3xHA; URA3 integration	this work
pKW6644	pMLP1-SV40NLS-Mlp2-N+NBD(aa1-600)-3xHA; URA3 integration	this work
pKW6645	pMLP1-SV40NLS-Mlp2-NBD(aa354-600)-3xHA; LEU2; TRP1 integration	this work
pKW6646	pMLP1-SV40NLS-Mlp2-N+NBD(aa1-600)-3xHA; LEU2; TRP1 integration	this work
pKW6647	pMLP1-Pml39-Δ3-20-mNG; LEU2; TRP1 integration	this work
pKW6648	pMLP1-Pml39-Δ3-40-mNG; LEU2; TRP1 integration	this work
pKW6649	pMLP1-Pml39-Δ3-77-mNG; LEU2; TRP1 integration	this work
pKW6651	pMLP1-Pml39core(aa78-316)-mNG; LEU2; TRP1 integration	this work
pKW6652	pMLP1-Pml39-Δ3-20-3xHA; LEU2; TRP1 integration	this work
pKW6653	pMLP1-Pml39-Δ3-40-3xHA; LEU2; TRP1 integration	this work
pKW6654	pMLP1-Pml39-Δ3-77-3xHA; LEU2; TRP1 integration	this work
pKW6656	pMLP1-Pml39core(aa78-316)-3xHA; LEU2; TRP1 integration	this work
pKW6657	pMLP1-SV40NLS-Mlp2-dNBD-mNG; URA3 integration	this work
pKW6658	pMLP1-SV40NLS-Mlp2-dNBD-3xHA; URA3 integration	this work
pKW6659	pMLP1-SV40NLS-Mlp2-dNBD-3xHA; LEU2; TRP1 integration	this work
pKW6660	pMLP1-PML39Δ42-66-mNG; LEU2; TRP1 integration	this work
pKW6661	pMLP1-PML39Δ42-66-3xHA; LEU2; TRP1 integration	this work
pKW6662	pMLP1-PML39Δ317-end-mNG; LEU2; TRP1 integration	this work
pKW6663	pMLP1-PML39Δ317-end-3xHA; LEU2; TRP1 integration	this work
**Template Plasmids for PCR**		
pKW1008	pFA6a-Flag-kanMX6	Dultz et al., 2018
pKW2934	pFA6a-3V5-IAA17-NATMX	Zsok et al., 2024
pKW4983	pFA6a-link-SV40NLS-yEGFP-CaURA3	Zsok et al., 2024
pKW4945	pRITE_loxP_GFP_URA3_loxP_notag	Kralt et al., 2022
pKW1964	pFA6a-link-yEGFP-CaURA3	Sheff and Thorn et al. 2004
pKW5588	pFA6a-His3Mx6-ins3	Powers et al. 2022

Journal of Cell Science • Supplementary information

Table S3. Cell numbers analyzed in Quantifications

S1: RITE dot counting — 100 cells per condition and replicate

S1: total cell intensities

	KWY12605	KWY12606	KWY12842	KWY12843	KWY12371	KWY12372
MIC2035_7	174	36	29	56	176	176
MIC2035_8	517	284	107	293	337	312
MIC2035_9	461	187	103	67	474	464

S2B: total cell intensities

	KWY4374	KWY4391	KWY4380	KWY4393	KWY4419	KWY4420	KWY4417
MIC2038_1	n.d.	286	n.d	310	403	278	164
MIC2038_3	653	387	584	696	419	430	596
MIC2038_6	219	252	44	214	118	54	78

4A: 7h NPC intensity

	KWY10309	KWY12230	KWY10882	KWY4402
MIC2026_1	245	333	385	520
MIC2026_2	741	307	231	307
MIC2026_3	331	255	416	352

S4B: 0h NE intensity

	KWY10309	KWY12230	KWY10882	KWY4402
MIC2026_1	106	101	112	105
MIC2026_2	102	100	78	101
MIC2026_3	101	101	104	103

S4C: 7h NPC intensity

	KWY10750	KWY4404	KWY10998	KWY4403
MIC2026_1	251	75	149	n.d.
MIC2026_2	436	129	75	n.d.
MIC2026_3	412	116	45	n.d.

5E&5SC: Mlp1 in Pml39 mut

	KWY10882	KWY12931	KWY13020	KWY13021	KWY13022	KWY13024	KWY13038	KWY13039
MIC2094_1	50		50	50	50	48	52	49
MIC2094_2	50	50	53	53	51	62	62	62
MIC2094_3	51	63	63	51	52	51	63	50

Journal of Cell Science • Supplementary information
