# Supplementary material for: Mlp1 and Mlp2 cooperate to build a stoichiometric nuclear pore basket in budding yeast
Source: J Cell Sci. 2026 Jul 27;139(14):jcs264865. doi: 10.1242/jcs.264865 (PMC13490735; doi:10.1242/jcs.264865)
Supplement: Supplementary file 2 [file joces-139-264865_review_history.pdf]

Mlp1 and Mlp2 cooperate to build a stoichiometric nuclear pore basket in budding yeast

Keri Schmidt, Alexandra P. Schürch and Elisa Dultz
DOI: 10.1242/jcs.264865

Editor: Abigail Buchwalter

Review timeline
Original submission: 10 March 2026
Editorial decision: 14 April 2026
First revision received: 10 June 2026
Accepted: 12 June 2026

Original submission

First decision letter

MS ID#: jcs.264865

MS TITLE: Mlp1 and Mlp2 cooperate to build a stoichiometric nuclear pore basket in budding yeast

AUTHORS: Keri Schmidt; Alexandra P Schürch; Elisa Dultz

ARTICLE TYPE: Research Article

Dear Dr Dultz,

We have now reached a decision on the above manuscript.

As you will see, the reviewers gave favourable reports but raised some critical points that will require amendments to your manuscript. In particular, Reviewer 1 raised several possible ways to test the predictions of your model which could be addressed by further experiments, further AlphaFold modeling, and/or adjustments to the claims made in the Results and Discussion. I hope that you will be able to address the reviewers' critiques because I would like to be able to accept your paper, pending further comments from reviewers.

Reviewer 1

SUMMARY OF THE ADVANCE MADE IN THIS PAPER AND ITS POTENTIAL SIGNIFICANCE TO THE FIELD

In this study, Schmidt et al. investigate the biogenesis and maintenance of the nuclear basket, the nucleoplasmic extension of nuclear pore complexes, using genetics, imaging, and structural modeling in budding yeast. They specifically explore the reciprocal interactions underlying the recruitment of distinct nuclear basket components (Nup60, Mlp1, Mlp2 and Pml39). Their findings extend previous work by demonstrating the presence of Nup60-independent NPC-binding determinants within the C-terminus of Mlp1 (Figs. 1-2), identifying a role for Mlp2 in stabilizing Mlp1 anchoring (Fig. 3), and exploring how Mlp1 and Mlp2 contribute to Pml39 anchoring (Fig. 5). Building on their data and AlphaFold3 modelling, the authors propose a model of Mlp1-Mlp2-Pml39 interactions and stoichiometry within the nuclear basket (Fig. 4). Overall, the featured experiments are well controlled and analyzed, and the conclusions are thoughtfully discussed in the context of existing literature, providing interesting insights into NPC basket biogenesis. However, a number of additional experiments could be performed to further strengthen the authors' claims, test their

© 2026. Published by The Company of Biologists under the terms of the Creative Commons Attribution License (https://creativecommons.org/licenses/by/4.0/).

proposed model, and thus enhance the overall impact of the study. Given the tools already in place, further mechanistic insight could be obtained through relatively straightforward approaches.

SUGGESTIONS TO AUTHORS

Major comments

1. The authors demonstrate that determinants mediating Nup60-independent association of Mlp1 with NPCs reside in its C-terminus (Fig. 2).
- One possibility is that Mlp1 engages in direct interactions with scaffold nucleoporins that facilitate its Nup60-independent recruitment. Crosslinking data from Kim et al. (2018) suggest additional contacts of Mlp1 (and Mlp2) with components of the inner ring (e.g., Nup157), the outer ring, and Nsp1. Could the authors test potential candidates for such interactions, either by AF3 modelling, or by inactivating these respective Nups in the context of Nup60 depletion?
- Alternatively, the gradual increase in NPC localization observed with longer Mlp1 truncations (Fig. 2D) may reflect recruitment mediated by heterodimerization with another basket component, e.g Mlp2. Could the authors check whether Nup60-independent Mlp1 association to NPCs requires Mlp2, by combining Nup60 depletion with MLP2 inactivation, or by testing an Mlp2 mutant protein lacking its NBD?

2. The AF3 model depicted in Fig. 4 is only partially supported by the experimental data, which do not formally prove the concomitant association of Mlp1 and Mlp2 homodimers with Pml39. In addition, while the stoichiometry of Mlp1, Mlp2 and Pml39 in the proposed basket model is in line with previous quantifications of GFP-tagged proteins at the NE performed by the authors (Zsok et al, 2024), it seems at odds with earlier work (Kim et al, 2018), which reported one copy each of Mlp1 and Mlp2 per NPC spoke in Mlp1 affinity-purified complexes. An alternative model could therefore be considered, in which a single Mlp1-Mlp2 proximal heterodimer mediates Pml39 recruitment. This would be consistent with the reciprocal dependence of Mlp1 and Mlp2 for NPC association (Fig. 3D and 5D), as well as with the loss of Pml39 scored in both mlp1 and mlp2 mutants (Fig. 5C-D).
- Could the authors test whether the putative Mlp2 NBD is sufficient for NPC association? Does expression of a truncated Mlp2 version containing only the NBD prevent Pml39 recruitment (mirroring the phenotype scored in Fig. 5D with Mlp1 NBD)?
- The AF3 model predicts that the Mlp2 dimer binds in between the lobes of Pml39, while Mlp1 associates to an adjacent site. Could the authors introduce structure-guided point mutations within these two distinct binding sites and further assess their impact on Pml39 recruitment?
- Could the authors model the interaction of Pml39 with an unique Mlp1-Mlp2 heterodimer (1:1:1 stoichiometry)?

3. The AF3 model further suggests that Pml39 recruits the distal Mlp1 population through its N and C terminal helixes. To test this, the authors use a 'core' Pml39 truncation construct lacking these domains, indeed resulting in decreased Mlp1 signals at NPCs (Fig 5E). However, this truncated protein, while still present at NPCs, also shows partial cytoplasmic mislocalization, complicating the interpretation of how much of it is functionally associated with the NE.
- Could the authors use their AF3 modeling to design point mutations in Pml39 N- and C-ter helixes that could similarly interfere with Mlp1 NBD binding, without affecting protein expression or localization? This would considerably strengthen the model in which distinct Pml39 interfaces are used to bind proximal and distal Mlp1 populations.
- Could the authors model the interaction between the Mlp2 NBD and Pml39? The prediction would be that while able to bind Nup60, the putative Mlp2 NBD cannot interact with Pml39 N- and C- ter helixes, thus explaining why only Mlp1 would contribute to the distal filament population.

Minor comments

1. Introduction p2: "the Sumo-protease Ulp1, which is implicated in a variety of functions including RNA processing…". To this reviewer's knowledge, there is no direct evidence supporting a role for Ulp1 in RNA processing.
2. Fig. 2 and p4 - "the longest (Mlp1) constructs (aa 1-1779 and full-length) retained NPC localization but formed additional bright nucleoplasmic foci". Are these constructs mildly overexpressed as compared to shorter truncations?

© 2026. Published by The Company of Biologists under the terms of the Creative Commons Attribution License (https://creativecommons.org/licenses/by/4.0/).

Reviewer 2

SUMMARY OF THE ADVANCE MADE IN THIS PAPER AND ITS POTENTIAL SIGNIFICANCE TO THE FIELD

In this manuscript, Schmidt et al. refine the model of how the nuclear basket is assembled and the authors address which interactions are important for assembling Mlp1, Mlp2, and Pml39, independent of Nup60. The structure of the basket of NPCs is the least understood subcomplex of NPCs and refining its structure is very valuable for the field. The authors present a well-controlled set of experiments, employing deletion/degron and truncation strains and the doRITE assay for visualizing NPCs. New findings include that Mlp1 can bind to the NPC via the C-terminal coiled-coil domain, and that Mlp2 can interact with the pore independently. Together with Mlp1 it recruits Pml39, which can bind an additional copy of Mlp1. These latter refinements are based on interaction models predicted by AlphaFold, leading to interaction predictions that were subsequently tested by additional experiments. This further refines the model of how the distal ring of Mlp2 is built via interactions with Pml39.

SUGGESTIONS TO AUTHORS

Minor comments
Overall this represents a solid set of data that is valuable and should be published. However, before acceptance, the manuscript would benefit from a round of clarifications/corrections. Throughout the manuscript, there are a number of points that are incorrect/incomplete/unclear listed in detail below (e.g. missing references and missing/wrong references to figures) that need to be corrected.

A main point for clarification is the use of various derivatives of Mlp1 (and its truncations): i.e constructs without an extra NLS, with an N-terminal NLS, or with an NLS-GFP at the C-terminus. In few cases it is written that the NLS is a SV40NLS but in most cases it is not stated which NLS was used. Can the authors please address: Is an NLS really required in these studies? Can the authors clarify the different uses of the NLSs? Can the authors exclude that it might interfere with interactions in the basket? Could it enforce NPC association? Or alternatively, since an NLS may have a transport factor bound, could this interfere with interactions in the basket, particularly given that release of the karyopherin from the NLS takes place at the basket? These questions should be discussed to clarify the choices and address the uncertainties.

Specific, textual comments and questions are listed below:
Comments, throughout the main text:
1. Title and Abstract:
Title: Also Pml39 plays a crucial role, to build the distal ring, maybe that is worth mentioning in the title?

2. Introduction:
- Line 25: The mention of the "stack of three rings" would benefit from a schematic of the nuclear pore complex (NPC), highlighting the three rings. Such a figure would also facilitate discussion of the double nuclear ring, which may explain why both Mlp1 and Mlp2 bind the ring (see discussion, line 265). It may be clearer to state "that form the scaffold of the central channel."
- Line 26: Please clarify which "compartments" are referred to.
- In general: Consider expanding the introduction with more information on the current model of the nuclear basket, especially since the paper seeks to refine it. Figure 1A helps clarify the structure, but it's unclear which components of this model are already accepted (e.g., the second copy of Mlp1 in the distal ring, its relation to Pml39). Is the arrangement in Figure 1A your refined model or is it based on existing models? Also, Nup1 and Nup2 are depicted but are not central to your study.

3. Results Section:
Line 113: is it known why Mlp1 is more stable at 23 degrees in a Nup60delta (i.e less dependent on Nup60 to be built in the pore at lower temperatures)?
Lines 112-129: Upon Nup60 deletion/depletion, Mlp1 and N+NBD-GFP form a single bright focus. The possible involvement of Pml39 is discussed, but do the authors have hypotheses about the mechanism behind this single focus formation?

© 2026. Published by The Company of Biologists under the terms of the Creative Commons Attribution License (https://creativecommons.org/licenses/by/4.0/).

Lines 125 onward: a bright focus for N+NBD is observed, which the authors attribute to the binding to Pml39; Did the authors test this in a Pml30delta?
Line 138: Please add a reference for the Nab2 binding site.
Line 152: Reference to Figure S1B is missing and should be included, as it provides information about Mlp1 expression levels.
Line 156: A reference to Figure 3B is missing.
Line 164: single NPC foci: how do we know that these big foci indeed contain NPCs and not only MLP1?
Line 166 (and elsewhere): The term "uninduced mlp2delta cells" needs clarification. Is this referring to cells that have not undergone the RITE procedure?
Line 171: Here, the authors seem to refer to the anchoring of the same extra pool of Mlp1. Please clarify.
Line 174: Additional references are needed.
Line 191: The claim that Mlp1 recruits a dimer of Mlp2 is not confirmed in lines 205 and further, right? Please update the model proposed in line 190-191.
Lines 209-211: just out of interest: does Mlp2 localize to the NPC when Nup60 is deleted or depleted?
Line 211: "Mlp2 localizes to the NPC…" Please refer explicitly to the relevant figure.
Line 212-213: you mean the NBD regio of Mlp2, right?
Lines 227/228: The mention of Nup133delta—please clarify its relevance here.
Line 226: did you also test single mutants, meaning only the N-terminus or C-terminus of Pml39 deleted? This to conform that both short helices in Pml39 are needed.
Last paragraph of the results section: Pml39 core is required for binding additional Mlp1 pool to the pore (as shown in 5E), but explicit mention of the 50% reduction is lacking.

4. Discussion Section:
Line 248: If previous models are not correct, clearly state what was incorrect, what your paper corrects, and which aspects are refined by your results. Was the earlier model based on erroneous data or misinterpretation? Please elaborate and provide supporting information.
Lines 251-253: do you mean extending the C-terminal domain even further than in the WT Mlp1?
Lines 255/256: Please further substantiate the discussion here and cite relevant papers.
Lines 258 and following: What do the results from Zsok et al. show? Their paper proposes the basket has 16 copies of Mlp1, 8 of Mlp2, and 4 of Pml39. Your manuscript doubles this (keeping the ratios). On what is the doubling of the numbers based and why was it different in Zsok et al? Please clarify and introduce stoichiometry/rationale in the introduction as well, since these ratios were established before.
Line 269: Figure 4c should actually be Figure 5; Figure 4c does not exist.
Line 285: "distal ring"—the manuscript would benefit from a schematic; it is currently unclear what/where this ring is.
Line 308: The word "exact"—what data support the proposed stoichiometry (32-16-8) such that you now claim it as exact?
Line 322/323: "not affected by it"—explain why this is the case.

5. Comments to the figures:
Figure 1:
The doRITE technique is well explained, but in Figure 1C (bottom), the dark green is difficult to distinguish.
The question remains whether "dim foci" indicate that released Mlp1 is rapidly degraded, or whether they reflect single NPC pores while bright foci might actually represent multiple pores that are close together. The microscope's resolution may limit interpretation.
Figure 1D shows a 16-hour time point, while S1A shows a 15-hour point. Please correct for consistency.
Figure 1D, but this comment actually applies to all figures and was mentioned as a main concern: Not all constructs have an NLS; some include an N-terminal SV40 NLS, others an NLS-GFP at the C-terminus. The influence of NLS location/presence is not sufficiently discussed, yet it may affect the results. Please indicate in the figures and/or in the text when an NLS is added, which NLS and if it's N-terminal or C-terminal. Am I correct that in this figure no NLS is added?
Add the quantifications from the microscopy of 1D (= figure S1A).
Figure 2A: I guess the grey triangle means Nup60 is deleted? In Figure 1A Nup60 also has a tail; why is this not cartooned in grey Am I right to assume that the Nucleoplasmic localization of e.g. Mlp1-

© 2026. Published by The Company of Biologists under the terms of the Creative Commons Attribution License (https://creativecommons.org/licenses/by/4.0/).

NBD in 2C is caused by the presence of the NLS? From this figure I would conclude that the C-terminus of Mlp1 is required but not fully sufficient for Nup60-independent NPC localization, since the big foci are formed.
In panel D: In the text it is stated that the NPC signal strengthens progressively as the C-termnal domain is extended—this is difficult to see from the microscopy pictures? Can the authors include a quantification of this?

Figure 3:
When MLP2 is deleted, the FL Mlp1 signal halve (3B); C-terminal truncations already show half the intensity in WT. The same can be seen in Fig 3C and 3Dand in 5E. I guess we are looking here at the loss of Mlp1 in the distal ring? Maybe corroborate on this further, since it fully confirms the model and stoichiometry of Mlp1 to Mlp2/Pml39.
In figures 3B NPC intensity shown and in 3C NE intensity: why these differences?
The title of the Figure legend might suggest that Mlp2 anchors all Mlp1 to the NPC. Revise legend to clarify that only half of Mlp1 (i.e. the distal ring Mlp1) depends on Mlp2, not all.

Figure 4:
The model in panel B does not fully match the AlphaFold predictions in 4A (blue and orange helices are oriented differently).
Is panel B the final model you propose, should it be moved to Figure 5?

Supplement: strain table
A set of strains in this table (Mlp1 truncations with an mCherry, e.g. KWY12876, is in the table but not used in any of the experiments. It seems that this set of strains is used for colocalization of Pml39, Mlp1 and Mlp2 but did not make it into the paper?

First revision

Author response to reviewers' comments

Response to reviewers: Our answers to the reviewers comments are shown in blue. Verbatim citations from the manuscript are shown in purple.

Reviewer 1: SUMMARY OF THE ADVANCE MADE IN THIS PAPER AND ITS POTENTIAL SIGNIFICANCE TO THE FIELD

In this study, Schmidt et al. investigate the biogenesis and maintenance of the nuclear basket, the nucleoplasmic extension of nuclear pore complexes, using genetics, imaging, and structural modeling in budding yeast. They specifically explore the reciprocal interactions underlying the recruitment of distinct nuclear basket components (Nup60, Mlp1, Mlp2 and Pml39). Their findings extend previous work by demonstrating the presence of Nup60-independent NPC-binding determinants within the C-terminus of Mlp1 (Figs. 1-2), identifying a role for Mlp2 in stabilizing Mlp1 anchoring (Fig. 3), and exploring how Mlp1 and Mlp2 contribute to Pml39 anchoring (Fig. 5). Building on their data and AlphaFold3 modelling, the authors propose a model of Mlp1-Mlp2-Pml39 interactions and stoichiometry within the nuclear basket (Fig. 4).
Overall, the featured experiments are well controlled and analyzed, and the conclusions are thoughtfully discussed in the context of existing literature, providing interesting insights into NPC basket biogenesis. However, a number of additional experiments could be performed to further strengthen the authors' claims, test their proposed model, and thus enhance the overall impact of the study. Given the tools already in place, further mechanistic insight could be obtained through relatively straightforward approaches.

We thank the reviewer for their positive assessment and the good suggestions for additional experiments, which have helped us to strengthen our paper.

SUGGESTIONS TO AUTHORS

© 2026. Published by The Company of Biologists under the terms of the Creative Commons Attribution License (https://creativecommons.org/licenses/by/4.0/).

Major comments

1. The authors demonstrate that determinants mediating Nup60-independent association of Mlp1 with NPCs reside in its C-terminus (Fig. 2).

- One possibility is that Mlp1 engages in direct interactions with scaffold nucleoporins that facilitate its Nup60-independent recruitment. Crosslinking data from Kim et al. (2018) suggest additional contacts of Mlp1 (and Mlp2) with components of the inner ring (e.g., Nup157), the outer ring, and Nsp1. Could the authors test potential candidates for such interactions, either by AF3 modelling, or by inactivating these respective Nups in the context of Nup60 depletion?
- Alternatively, the gradual increase in NPC localization observed with longer Mlp1 truncations (Fig. 2D) may reflect recruitment mediated by heterodimerization with another basket component, e.g Mlp2. Could the authors check whether Nup60-independent Mlp1 association to NPCs requires Mlp2, by combining Nup60 depletion with MLP2 inactivation, or by testing an Mlp2 mutant protein lacking its NBD?

We thank the reviewer for suggesting these targeted experiments. Indeed, we could show that the maintenance of Mlp1 at the NPC upon depletion of Nup60 relies on Mlp2 and requires both the NBD of Mlp2 and its coiled-coil domains. This suggests that lateral interactions between Mlp1 and Mlp2 in combination with the interaction interfaces of each of these proteins with the NPC scaffold can support basket stability. We have included this new dataset in the new Figure 3C-D.

2. The AF3 model depicted in Fig. 4 is only partially supported by the experimental data, which do not formally prove the concomitant association of Mlp1 and Mlp2 homodimers with Pml39. In addition, while the stoichiometry of Mlp1, Mlp2 and Pml39 in the proposed basket model is in line with previous quantifications of GFP-tagged proteins at the NE performed by the authors (Zsok et al, 2024), it seems at odds with earlier work (Kim et al, 2018), which reported one copy each of Mlp1 and Mlp2 per NPC spoke in Mlp1 affinity-purified complexes. An alternative model could therefore be considered, in which a single Mlp1-Mlp2 proximal heterodimer mediates Pml39 recruitment. This would be consistent with the reciprocal dependence of Mlp1 and Mlp2 for NPC association (Fig. 3D and 5D), as well as with the loss of Pml39 scored in both mlp1 and mlp2 mutants (Fig. 5C-D).

- Could the authors test whether the putative Mlp2 NBD is sufficient for NPC association? Does expression of a truncated Mlp2 version containing only the NBD prevent Pml39 recruitment (mirroring the phenotype scored in Fig. 5D with Mlp1 NBD)?
- The AF3 model predicts that the Mlp2 dimer binds in between the lobes of Pml39, while Mlp1 associates to an adjacent site. Could the authors introduce structure-guided point mutations within these two distinct binding sites and further assess their impact on Pml39 recruitment?
- Could the authors model the interaction of Pml39 with an unique Mlp1-Mlp2 heterodimer (1:1:1 stoichiometry)?

We thank the reviewer for correctly pointing out this point of uncertainty. We were not able to fully solve this question in the current work, but we have added some additional data to understand Pml39 recruitment to the NPC and modified the presentation to present the two options (heterodimer versus two homodimers) in a more balanced way.
Experimentally, we better characterized which domains of Mlp1 and Mlp2 are required for Pml39 recruitment. We now show in Figure 5C, D that for both Mlp1 and Mlp2 the NBD is not sufficient for Pml39 recruitment while the N-terminus is necessary and sufficient. Furthermore, we now only show the interactions of Pml39 with each homodimer individually in the main figure (Figure 5B) while we moved the model of all Mlp1/2 homodimers interacting with Pml39 to the supplement (Figure S5B), were we now also show models of heterodimers in interaction with Pml39 (Figure S5A). The heterodimers have similar confidence values as the homodimers. Further dissection of this interaction network may better be addressed by structural studies and go beyond our current work.

3. The AF3 model further suggests that Pml39 recruits the distal Mlp1 population through its N and C terminal helixes. To test this, the authors use a 'core' Pml39 truncation construct lacking these domains, indeed resulting in decreased Mlp1 signals at NPCs (Fig 5E). However, this truncated protein, while still present at NPCs, also shows partial cytoplasmic mislocalization, complicating the interpretation of how much of it is functionally associated with the NE.

- Could the authors use their AF3 modeling to design point mutations in Pml39 N- and C-ter helixes that could similarly interfere with Mlp1 NBD binding, without affecting protein expression

© 2026. Published by The Company of Biologists under the terms of the Creative Commons Attribution License (https://creativecommons.org/licenses/by/4.0/).

or localization? This would considerably strengthen the model in which distinct Pml39 interfaces are used to bind proximal and distal Mlp1 populations.
- Could the authors model the interaction between the Mlp2 NBD and Pml39? The prediction would be that while able to bind Nup60, the putative Mlp2 NBD cannot interact with Pml39 N- and C- ter helixes, thus explaining why only Mlp1 would contribute to the distal filament population.
We agree with the reviewer that the change in expression level we observed for the Pml39core construct was somewhat problematic. We now added experiments with several constructs carrying shorter truncations (Figure 5E, S5C). While all truncations touching helices predicted to interact with the Mlp1-NBD affected Mlp1 recruitment, some of these constructs did not show strongly increased expression levels. A truncation that removed a loop in the N-terminus that was not predicted to participate in the interaction with Mlp1-NBD did not affect Mlp1 recruitment, further verifying the interaction mode predicted by AF3. The different truncations also suggest that it is the deletion of residues 20-40 which causes the overexpression phenotype of some mutants. Possibly this region contains a degron or promotes nuclear localization, since wild-type Pml39 is enriched in the nucleus when unable to associate with the NPC.
We also modeled the interaction between the Mlp2 NBD and Pml39, which we now show in Figure S5A. AF3 predicts an interaction analogous to the one predicted for the Mlp1 NBD with similar confidence. The two proteins are highly similar and the prediction does not provide a basis to understand why the interaction with the Mlp2 NBD does not seem to occur in cells.

Minor comments

1. Introduction p2: "the Sumo-protease Ulp1, which is implicated in a variety of functions including RNA processing…". To this reviewer's knowledge, there is no direct evidence supporting a role for Ulp1 in RNA processing.
We agree that there is no evidence for direct involvement. We have removed this statement.

2. Fig. 2 and p4 - "the longest (Mlp1) constructs (aa 1-1779 and full-length) retained NPC localization but formed additional bright nucleoplasmic foci". Are these constructs mildly overexpressed as compared to shorter truncations?
We have added a quantification of the total cell fluorescence intensity in Figure S2B to assess expression levels. Apart from the construct 1-1585, the other constructs are all expressed at similar levels. We therefore think it is not very likely that expression levels are the main driver of focus formation. That said, we know from other experiments that most fragments can form foci if overexpressed and expression levels can therefore very well play a role. We mention this possibility in the text now.

Reviewer 2: SUMMARY OF THE ADVANCE MADE IN THIS PAPER AND ITS POTENTIAL SIGNIFICANCE TO THE FIELD

In this manuscript, Schmidt et al. refine the model of how the nuclear basket is assembled and the authors address which interactions are important for assembling Mlp1, Mlp2, and Pml39, independent of Nup60. The structure of the basket of NPCs is the least understood subcomplex of NPCs and refining its structure is very valuable for the field. The authors present a well-controlled set of experiments, employing deletion/degron and truncation strains and the doRITE assay for visualizing NPCs. New findings include that Mlp1 can bind to the NPC via the C-terminal coiled-coil domain, and that Mlp2 can interact with the pore independently. Together with Mlp1 it recruits Pml39, which can bind an additional copy of Mlp1. These latter refinements are based on interaction models predicted by AlphaFold, leading to interaction predictions that were subsequently tested by additional experiments. This further refines the model of how the distal ring of Mlp2 is built via interactions with Pml39.

SUGGESTIONS TO AUTHORS

Minor comments

Overall this represents a solid set of data that is valuable and should be published. However, before acceptance, the manuscript would benefit from a round of clarifications/corrections. Throughout the manuscript, there are a number of points that are incorrect/incomplete/unclear

© 2026. Published by The Company of Biologists under the terms of the Creative Commons Attribution License (https://creativecommons.org/licenses/by/4.0/).

listed in detail below (e.g. missing references and missing/wrong references to figures) that need to be corrected.

A main point for clarification is the use of various derivatives of Mlp1 (and its truncations): i.e constructs without an extra NLS, with an N-terminal NLS, or with an NLS-GFP at the C-terminus. In few cases it is written that the NLS is a SV40NLS but in most cases it is not stated which NLS was used. Can the authors please address: Is an NLS really required in these studies? Can the authors clarify the different uses of the NLSs? Can the authors exclude that it might interfere with interactions in the basket? Could it enforce NPC association? Or alternatively, since an NLS may have a transport factor bound, could this interfere with interactions in the basket, particularly given that release of the karyopherin from the NLS takes place at the basket? These questions should be discussed to clarify the choices and address the uncertainties.
Thank you for pointing out our omission to explain the necessity for an NLS in some of our constructs. The endogenous NLS of Mlp1 is located in the C-terminal region. Since many of our truncation constructs lack this region, we incorporated the exogenous SV40NLS to achieve nuclear targeting and we also incorporated these in our full-length control constructs. We used the SV40NLS in all constructs and the nomenclature was homogenized in the revised strain table. In integration constructs, the NLS was fused to the N-terminus, but where the endogenous protein was tagged C-terminally directly in the genome, the NLS is also located in the C-terminus. Since in each case the full-length control protein is tagged in the same manner and recapitulates the behavior of the protein without an NLS, we are confident that the presence of the NLS does not affect our conclusions. This said, we can of course not exclude that the exogenous NLS may change the behavior of the protein in subtle ways, the same applies for the fluorescent and epitope tags we use. We have added a brief explanation of the need for an NLS in our constructs in the Methods Section. It reads: »The nuclear localization sequences of Mlp1 and Mlp2 are located in their C-terminal region (Strambio-de-Castillia et al., 1999). Since many of our truncation constructs thus lack an NLS, we added an SV40NLS in most constructs of Mlp1 and Mlp2 including the respective full-length controls. The presence of an exogenous NLS is in each case indicated in the strain information in Table S1.

Specific, textual comments and questions are listed below:

Comments, throughout the main text:
1. Title and Abstract:
Title: Also Pml39 plays a crucial role, to build the distal ring, maybe that is worth mentioning in the title?
Thank you for this suggestion. However, we decided to keep our previous title, because we want to emphasize the role of Mlp2 which was previously underappreciated in the field.

2. Introduction:
- Line 25: The mention of the "stack of three rings" would benefit from a schematic of the nuclear pore complex (NPC), highlighting the three rings. Such a figure would also facilitate discussion of the double nuclear ring, which may explain why both Mlp1 and Mlp2 bind the ring (see discussion, line 265). It may be clearer to state "that form the scaffold of the central channel."
We have added a schematic of the entire NPC in Figure 1A.

- Line 26: Please clarify which "compartments" are referred to.
This is clarified in the following sentence that explains the cytoplasmic and nucleoplasmic functions. We have added a colon for better clarity.

- In general: Consider expanding the introduction with more information on the current model of the nuclear basket, especially since the paper seeks to refine it. Figure 1A helps clarify the structure, but it's unclear which components of this model are already accepted (e.g., the second copy of Mlp1 in the distal ring, its relation to Pml39). Is the arrangement in Figure 1A your refined model or is it based on existing models? Also, Nup1 and Nup2 are depicted but are not central to your study.
We agree that Nup1 and Nup2 are not really relevant in the context of our study and have removed them from the schematic figure. Overall we have tried to describe the current state of knowledge in the field in the introduction, including the open questions some of which we

© 2026. Published by The Company of Biologists under the terms of the Creative Commons Attribution License (https://creativecommons.org/licenses/by/4.0/).

address in the study. To better illustrate the level of current understanding also in the figure, we have refined it to differentially depict interactions characterized in molecular detail by solid lines and interactions only demonstrated biochemically by dashed lines. We feel that expanding the introduction with more detail would be tedious for most readers.

3. Results Section:
Line 113: is it known why Mlp1 is more stable at 23 degrees in a Nup60delta (i.e less dependent on Nup60 to be built in the pore at lower temperatures)?
The molecular mechanism behind this is not known. There may be a thermolabile interaction or assembly step that is stabilized by the presence of Nup60 or by lower temperatures.

Lines 112-129: Upon Nup60 deletion/depletion, Mlp1 and N+NBD-GFP form a single bright focus. The possible involvement of Pml39 is discussed, but do the authors have hypotheses about the mechanism behind this single focus formation?
It has previously been proposed that the ability of Pml39 to interlink Mlp1 molecules leads to focus formation in conditions where Mlp1 cannot assemble into a basket (e.g. nup60d, Gunkel et al. 2023). This is likely what is happening here as well after long term depletion of Nup60. Why some constructs that are retained at the NPC but still become interlinked form foci and others do not, we do not know. It could be distinct interactors like Nab2, but as reviewer 1 indicates, differences in expression levels might also contribute.

Lines 125 onward: a bright focus for N+NBD is observed, which the authors attribute to the binding to Pml39; Did the authors test this in a Pml30delta?
We did not test this, but it is a plausible explanation. We mention it as a possibility in the text. Since this aspect is not really relevant for the interaction that occurs at the NPC, we do not consider this a hypothesis worth further investigating at this point.

Line 138: Please add a reference for the Nab2 binding site.
Thank you for noticing this omission. The reference (Fasken et al. 2008) was added.

Line 152: Reference to Figure S1B is missing and should be included, as it provides information about Mlp1 expression levels.
The reference to the figure was inserted and the unchanged protein levels of Mlp1 upon deletion of *MLP2* are now mentioned in the text.

Line 156: A reference to Figure 3B is missing.
This figure was removed from the revised version.

Line 164: single NPC foci: how do we know that these big foci indeed contain NPCs and not only MLP1?
These foci are diffraction limited and we cannot interpret their size. We have characterized single NPC foci in doRITE extensively in our previous publication, including their intensity, mobility and inheritance patterns (Zsok et al. 2024) and these data support the interpretation that most foci correspond to single NPCs. We cannot exclude that a subset of foci corresponding to several NPCs.

Line 166 (and elsewhere): The term "uninduced mlp2delta cells" needs clarification. Is this referring to cells that have not undergone the RITE procedure?
We have clarified this by writing: “Prior to RITE induction, a subset of *mlp2Δ* cells …”

Line 171: Here, the authors seem to refer to the anchoring of the same extra pool of Mlp1. Please clarify.
Done.

Line 174: Additional references are needed.
Inserted.

Line 191: The claim that Mlp1 recruits a dimer of Mlp2 is not confirmed in lines 205 and further, right? Please update the model proposed in line 190-191.
This section of the paper was changed, so the comment does not apply anymore.

© 2026. Published by The Company of Biologists under the terms of the Creative Commons Attribution License (https://creativecommons.org/licenses/by/4.0/).

Lines 209-211: just out of interest: does Mlp2 localize to the NPC when Nup60 is deleted or depleted?
It does, as long as Mlp1 is there. This new data is now shown in Figure 3D.

Line 211: "Mlp2 localizes to the NPC…" Please refer explicitly to the relevant figure.
This is now shown in Figure 3B and referenced in the text.

Line 212-213: you mean the NBD regio of Mlp2, right?
Yes. We agree that this was formulated in a somewhat roundabout way. It is now explained in a different place in the text in a more direct way.

Lines 227/228: The mention of Nup133delta—please clarify its relevance here.
We mention this result here, because it shows that the Pml39 core construct retains some functionality of the full length protein.

Line 226: did you also test single mutants, meaning only the N-terminus or C-terminus of Pml39 deleted? This to conform that both short helices in Pml39 are needed.
We have now included this new data and show that each of the helices is needed (Figure 5D).

Last paragraph of the results section: Pml39 core is required for binding additional Mlp1 pool to the pore (as shown in 5E), but explicit mention of the 50% reduction is lacking.
We have now added the values for the relative reduction of Mlp1 intensity at the NE/NPC in the relevant figures (4A, S4B, S4C and 5E). The measured reduction is reproducibly higher than 50% (reduction to ~ 65%) as previously shown (Gunkel et al. 2023, Zsok et al, 2023). This could be because the background subtraction is not perfect due to the higher nucleoplasmic background in these cells. However, it could also be that the released pool is indeed less than 50%. We discuss this now in the Discussion.

4. Discussion Section:
Line 248: If previous models are not correct, clearly state what was incorrect, what your paper corrects, and which aspects are refined by your results. Was the earlier model based on erroneous data or misinterpretation? Please elaborate and provide supporting information.
We apologize that our statement here was not very precise, we thank the reviewer for catching this. The sentence should have read "[…]this hypothesis proved not to be correct […]" referring to the hypothesis that the NBD is sufficient for Mlp1 interaction in the absence of Nup60. This was now modified.

Lines 251-253: do you mean extending the C-terminal domain even further than in the WT Mlp1?
That is not what we meant to imply. However, due to the new findings on the role of Mlp2, this section in the text was now changed and does no longer include this ambiguous formulation.

Lines 255/256: Please further substantiate the discussion here and cite relevant papers.
Since we have now identified Mlp2 as responsible for the observed stabilization, this discussion point has become superfluous and was removed.

Lines 258 and following: What do the results from Zsok et al. show? Their paper proposes the basket has 16 copies of Mlp1, 8 of Mlp2, and 4 of Pml39. Your manuscript doubles this (keeping the ratios). On what is the doubling of the numbers based and why was it different in Zsok et al? Please clarify and introduce stoichiometry/rationale in the introduction as well, since these ratios were established before.
The results from Zsok et al. only determine ratios for the stoichiometry of the basket Nups, but not absolute numbers. In that paper, the minimal possible number of Nups based on this stoichiometry was proposed. Here, we proposed the double number because this seems to more plausibly explain our data as well as other more recent data (e.g. Obarska-Kosinska et al. 2026 preprint). Nevertheless, it is true that what we propose is only a model and this is clearly stated. Since an explicit model for nuclear basket architecture consistent with a 16:8:4 ratio for the basket Nups was not proposed, we prefer not to discuss this previous proposition in the introduction as it would be rather more confusing than helpful.

Line 269: Figure 4c should actually be Figure 5; Figure 4c does not exist.

© 2026. Published by The Company of Biologists under the terms of the Creative Commons Attribution License (https://creativecommons.org/licenses/by/4.0/).

Yes, thank you for spotting this. The figure reference was corrected.

Line 285: "distal ring"—the manuscript would benefit from a schematic; it is currently unclear what/where this ring is.
We have added a schematic depicting the entire NPC in Figure 1A.

Line 308: The word "exact"—what data support the proposed stoichiometry (32-16-8) such that you now claim it as exact?
Here, we refer to the open questions. The exact stoichiometry is still unclear, since although several lines of evidence support our model with a 32-16-8 stoichiometry, we have not directly determined the absolute number of each component at individual NPCs.

Line 322/323: "not affected by it"—explain why this is the case.
In RITE induced cells at the 7h timepoint there is no longer any significant free pool of fluorescent molecules and therefore no differences in the background signal that needs to be corrected for in our measurements without RITE induction. In this case small inaccuracies in the placement of measuring the nuclear envelope signal can be strongly affected by different levels of signal inside and outside the nucleus, which is the case particularly in mutants that lead to the dissociation of the analyzed proteins from the nuclear envelope.

5. Comments to the figures:
Figure 1:
The doRITE technique is well explained, but in Figure 1C (bottom), the dark green is difficult to distinguish.
Thank you, we have replaced the color with a lighter shade of green.

The question remains whether "dim foci" indicate that released Mlp1 is rapidly degraded, or whether they reflect single NPC pores while bright foci might actually represent multiple pores that are close together. The microscope's resolution may limit interpretation.
As mentioned above, we have thoroughly characterized this method in our previous paper to show that most of the foci have a similar intensity and show inheritance patterns consistent with single NPCs. In some cases, brighter foci do likely represent multiple pores that are close together, but statistically this number is expected to be low at the 7h timepoint we analyze. Indeed, due to the higher number of labelled NPCs in *mlp2Δ* cells (Figure S1A), we would there expect a higher number of NPCs localizing too close together t o resolve them. Furthermore, the intensity decrease in *mlp2Δ* cells can also be seen when measuring the total fluorescent intensity at the nuclear envelope prior to RITE induction.

Figure 1D shows a 16-hour time point, while S1A shows a 15-hour point. Please correct for consistency.
We apologize for this labeling inconsistency. The timepoint shown is 16h. This was corrected in Figure S1A.

Figure 1D, but this comment actually applies to all figures and was mentioned as a main concern: Not all constructs have an NLS; some include an N-terminal SV40 NLS, others an NLS-GFP at the C-terminus. The influence of NLS location/presence is not sufficiently discussed, yet it may affect the results. Please indicate in the figures and/or in the text when an NLS is added, which NLS and if it's N-terminal or C-terminal. Am I correct that in this figure no NLS is added?
We have included a discussion on the use of NLSs in the methods section. The presence and location of an NLS in each construct can be seen from the strain table.

Add the quantifications from the microscopy of 1D (= figure S1A).
Does the reviewer mean that Figure S1A should be moved into the main figure? We prefer to leave it in the supplement, since it is important to view it in context with Figure S1B.

Figure 2A: I guess the grey triangle means Nup60 is deleted? In Figure 1A Nup60 also has a tail; why is this not cartooned in grey
We now chose a lighter grey to more clearly indicate the absence of Nup60. We also now omit the tail of Nup60 in all figures, since it is not a feature needed in the context of this study.

© 2026. Published by The Company of Biologists under the terms of the Creative Commons Attribution License (https://creativecommons.org/licenses/by/4.0/).

Am I right to assume that the Nucleoplasmic localization of e.g. Mlp1-NBD in 2C is caused by the presence of the NLS? From this figure I would conclude that the C-terminus of Mlp1 is required but not fully sufficient for Nup60-independent NPC localization, since the big foci are formed.
As discussed, the presence of Nup60 is required for incorporation of newly synthesized Mlp1. The big foci observed in the longer constructs likely consist of newly synthesized Mlp1 that cannot be incorporated in NPCs. Our conclusion is that the C-terminus of Mlp1 is sufficient for maintaining Mlp1 at the NPC in the absence of Nup60, but it relies on Nup60 for assembly into new baskets.

In panel D: In the text it is stated that the NPC signal strengthens progressively as the C-termnal domain is extended—this is difficult to see from the microscopy pictures? Can the authors include a quantification of this?
Unfortunately the foci present in many of these strains make a good quantification very difficult. Since we consider this point not so important, especially in view of the new data we added on the role of Mlp2, we did not attempt quantifications but we removed the word “progressively” from the text to weaken our statement.

Figure 3:
When MLP2 is deleted, the FL Mlp1 signal halve (3B); C-terminal truncations already show half the intensity in WT. The same can be seen in Fig 3C and 3Dand in 5E. I guess we are looking here at the loss of Mlp1 in the distal ring? Maybe corroborate on this further, since it fully confirms the model and stoichiometry of Mlp1 to Mlp2/Pml39.
Thank you for pointing this out. Since we have removed this particular dataset for better clarity of the storyline, we can now no longer make this point.

In figures 3B NPC intensity shown and in 3C NE intensity: why these differences?
We use NE intensity measurements for cells that were not induced by RITE (Figure 3C) and NPC intensity after 7h of RITE induction (Figure 3B, 3D). Generally, we avoid using NE intensity measurements when comparing different constructs, since differences in their expression levels can easily affect such quantifications. The NPC intensity measurements at 7h are less affected by this, since soluble pools are diluted and only the protein bound at individual NPCs is measured.

The title of the Figure legend might suggest that Mlp2 anchors all Mlp1 to the NPC. Revise legend to clarify that only half of Mlp1 (i.e. the distal ring Mlp1) depends on Mlp2, not all.
Due to the reorganization of the figures, this comment does not apply anymore. The two relevant figures are now entitled: Figure 3: Mlp2 can interact with the NPC on its own but is stabilized there by Mlp1. and Figure 4: Mlp2 contributes to anchoring of Pml39 to the NPC.

Figure 4:
The model in panel B does not fully match the AlphaFold predictions in 4A (blue and orange helices are oriented differently).
The orientation of the helices was improved.

Is panel B the final model you propose, should it be moved to Figure 5?
The model figure is now a separate figure 6.

Supplement: strain table
A set of strains in this table (Mlp1 truncations with an mCherry, e.g. KWY12876, is in the table but not used in any of the experiments. It seems that this set of strains is used for colocalization of Pml39, Mlp1 and Mlp2 but did not make it into the paper?
Thank you for spotting this. These strains were indeed not used in the paper. The strain table was corrected.

Second decision letter

MS ID#: jcs.264865R1

© 2026. Published by The Company of Biologists under the terms of the Creative Commons Attribution License (https://creativecommons.org/licenses/by/4.0/).

MS Title: Mlp1 and Mlp2 cooperate to build a stoichiometric nuclear pore basket in budding yeast

Authors: Keri Schmidt; Alexandra P Schürch; Elisa Dultz

Article Type: Research Article

Dear Dr Dultz,

I am happy to tell you that your manuscript has been accepted for publication in Journal of Cell Science, pending standard publication integrity checks.

Reviewer 1

The authors have adequately addressed my previous comments by performing additional experiments and providing new analyses and discussion. The manuscript is now suitable for publication.
One minor issue remains: in the revised version of Figure S5B, part of the legend is obscured by the figure and should be made fully visible.

© 2026. Published by The Company of Biologists under the terms of the Creative Commons Attribution License (https://creativecommons.org/licenses/by/4.0/).
